# Increased RAB31 Expression in Cancer-Associated Fibroblasts Promotes Colon Cancer Progression Through HGF-MET Signaling

**DOI:** 10.3389/fonc.2020.01747

**Published:** 2020-09-23

**Authors:** Tang Yang, Huang Zhiheng, Wang Zhanhuai, Xiao Qian, Liu Yue, Ge Xiaoxu, Wei Jingsun, Zheng Shu, Ding Kefeng

**Affiliations:** ^1^Key Laboratory of Cancer Prevention and Intervention, Ministry of Education, Department of Colorectal Surgery and Oncology, The Second Affiliated Hospital, Zhejiang University School of Medicine, Hangzhou, China; ^2^Department of Otorhinolaryngology, The Second Affiliated Hospital of Zhejiang University School of Medicine, Hangzhou, China

**Keywords:** colon cancer, cancer associated fibroblast (CAF), hepatocyte growth factor (HGF), cancer progression, Ras-related protein RAB31

## Abstract

RAB family proteins participate in the dynamic regulation of cellular membrane compartments and are dysregulated in a variety of tumor types, which may alter the biological properties of cancer cells such as proliferation, migration, and invasion. In our previous study, we found that Ras-related protein Rab-31 (RAB31) expression was increased in late-stage colorectal cancer (CRC). The role of RAB31 has never been investigated in CRC. In this study, we found that expression of RAB31 in the tumor stroma but not cancer cells of colon cancer predicted poor survival. RAB31 can be detected in primary cancer-associated fibroblasts (CAFs) and paired normal fibroblasts. Conditioned medium (CM) from RAB31 overexpressing CAFs significantly promoted migration of colon cancer cell lines *in vitro* and *in vivo*. This process may be mediated by paracrine action of hepatocyte growth factor (HGF), which was increased in the CM of RAB31-overexpressing CAFs. Blockade of HGF/MET signaling by drug inhibition, knockdown of mesenchymal to epithelial transition factor (MET) in RKO, or antibody neutralization of HGF abolished migration of RKO cells mediated by RAB31 expression in CAFs. We propose that in colon cancer, increased RAB31 expression in CAFs may contribute to tumor progression by regulating the secretion of HGF in the tumor stroma.

## Introduction

Colorectal cancer (CRC) is the third most common malignant neoplasm worldwide with over 1 million cases diagnosed every year (1). Over the last decade, with advances in sequencing techniques, huge efforts have been made in unraveling the molecular complexity behind CRC initiation and progression. In our earlier study, we combined ITRAQ mass spectrometry with gene microarray to screen for differentially expressed genes associated with CRC progression. RAB31 was found to be upregulated in stage IV CRC (2); however, its role in CRC progression has never been investigated.

RAB31 is a member of the RAB family proteins, an important subgroup of the GTPase superfamily (3, 4) which participates in the dynamic regulation of cellular membrane compartments and is implicated in membrane trafficking, Golgi complex organization, and the sorting and delivery of secretory/membrane proteins (5). RAB31 is primarily localized in the Golgi complex and can be found in endosomes and tubulovesicular structures originating from the Golgi complex (6). RAB31 has been reported to mediate the transport of the cation-dependent mannose-6-phosphate (CD-MPR) from the trans-Golgi network (TGN) to endosomes (7). The underlying mechanism may involve interaction between RAB31 and the Lowe oculocerebrorenal syndrome protein OCRL-1 (an inositol polyphosphate 5-phosphatase), which is required for TGN organization and transport carrier formation (8). Accumulating evidence has shown that altered RAB protein expression is associated with cancer progression (9). RAB31 has been reported to be associated with the malignant behavior of breast cancer, hepatocellular carcinoma, and gastric cancer (10–12). Increased RAB31 expression is significantly associated with distant metastasis-free survival and overall survival rate in breast cancer (13). Breast cancer cell lines with RAB31 overexpression *in vitro* switch from an invasive to a more proliferative phenotype (10), suggesting a pro-tumorigenic role for this protein in the respective cancer types.

While the molecular biology of cancer cells has been extensively studied, the tumor microenvironment (TME) also plays an important role in tumor progression. The TME, also known as the tumor stroma, consists of connective tissue and a variety of non-cancer cell types. Immune cells such as macrophages, cancer-associated fibroblasts (CAFs), and lymphocytes residing in the stroma have been shown to exhibit bidirectional effects on tumor progression (14–16). The tumor stroma is associated with prognosis in a variety of cancer types (17–21). In CRC, the tumor–stroma ratio predicts recurrence in patients treated with neoadjuvant chemotherapy (22, 23) and may serve as a prognosis factor in stage II CRC (24). According to the consensus molecular subtypes of classification for CRC (CMS classification), the CMS4 group, defined as a subtype of CRC with increased CAF component and mesenchymal activation, features higher resistance to chemotherapy and anti-EGFR therapy and has the worst prognosis (25). An important mechanism underlying TME-mediated tumor progression is through paracrine action. CAF is a heterogeneous group of fibroblast-like cells and a major component of the TME stromal cells. CAF secretion of growth factors such as EGF, FGF, HGF, TGF-β, and cytokines such as IL6 facilitates the migration of cancer cells and confers resistance to antitumor therapy (26, 27). CAFs can also promote tumor invasion and metastasis through physical contact (28). The pro-tumor functions of other stromal cells have also been widely studied (29, 30).

Since high-throughput screening based on bulk tissue does not discriminate cancer cells from stromal cells, further study is needed for better interpretation of the acquired results from our previous study. In this study, we aim to characterize the functional and clinical roles of RAB31 in the progression of colon cancer. We found that while RAB31 may be expressed in both cancer cells and stromal cells, stromal RAB31 expression is associated with poor prognosis in colon cancer. Overexpression of RAB31 in CAFs promoted colon cancer cell migration in an HGF/MET-dependent manner, suggesting a role for CAF-expressed RAB31 in the migration of colon cancer cells through regulating paracrine secretion of HGF. These results provide novel information on the role of RAB31 in colon cancer progression.

## Materials and Methods

### Patients

Paraffin-embedded colon tumor tissue samples were collected from the Second Affiliated Hospital of Zhejiang University, School of Medicine. A cohort of 98 patients receiving surgery from 2006 to 2007 was included. Patients who received neoadjuvant chemotherapy or incomplete surgical resection were excluded from the cohort. The diagnosis of adenocarcinoma in the patients was confirmed by two pathologists. One millimeter-diameter cores from each tumor were taken from the formalin-fixed paraffin-embedded tissues for the generation of tissue microarray.

### CMS Classification

Data for analysis of RAB31 expression and CMS classification including normalized gene expression data, CMS subtyping calls, and sample annotation were readily provided and downloaded through the Synapse platform (doi: 10.7303/syn2623706) (25). The data was sorted in Microsoft Excel, and statistical analysis and plotting were performed in GraphPad Prism 8.

### Immunohistochemistry

Paraffin-embedded tissues were collected to generate a tissue microarray. Immunohistochemistry was performed on 5-μm-thick sections. Briefly, the microarray sections were dewaxed in an oven at 60°C, deparaffinized in xylene, and rehydrated in graded alcohol. Heated TRIS-EDTA was used for antigen retrieval. Five percentage BSA was used to block non-specific binding. The slides were stained with primary antibodies in 5% BSA at a dilution of 1:200, rinsed in PBS three times, and incubated with secondary fluorescent antibodies followed by 3 more rinses in PBS. Assessments were independently conducted by 2 pathologists. The expression of RAB31 was graded in both the tumor cell and tumor stromal compartments using a 3-tier scale: 1, no staining or weak staining; 2, moderate staining; 3, strong staining. The results were then divided into negative staining, grade 1; and positive staining, grade 2 and 3 for further statistical analysis.

For immunostaining of cells, fibroblasts were culture on round glass coverslips in 12-well culture plates. Before staining, the coverslips were rinsed in PBS 3 times and fixed with 4% paraformaldehyde for 15 min. The following blocking and staining procedures were the same as described above.

### Cell Culture

All CRC cell lines were purchased from ATCC. HCT116 and HT29 were maintained in McCoy's 5A modified medium; RKO and DLD1 were maintained in RPMI-1640 medium; SW620 and SW480 were maintained in Leibovitz L-15 medium; LOVO, T84, and CACO2 were maintained in Dulbecco's Modified Eagle Medium. All culture mediums were supplemented with 10% fetal bovine serum (FBS, Gibco/Invitrogen, Carlsbad, CA, USA). SW480 and SW620 were cultured in a CO_2_-free incubator, while all other cells were maintained in a humidified incubator with 95% air and 5% CO_2_. The culture medium was refreshed every 3 days, and the cells were passed at 90% confluency.

### Preparation of Primary Fibroblast Cells and Fibroblast Conditioned Medium (CM)

Mucosa from tumor and adjacent normal tissue were obtained from freshly surgically resected samples and transferred to the lab in PBS containing 10% Povidone iodine within 30 min for further procedures. The tissues were rinsed in PBS containing 500 U/ml streptomycin and penicillin for 3 times, minced with surgical scissors into 2–4-mm^3^ chunks, and plated in a 60-mm culture dish in RPMI 1640 containing 10% FBS, 100 U/ml streptomycin, and penicillin and 2.5 μg/ml Amphotericin B. To ensure adherence to the culture dish, the culture medium must not submerge the tissue blocks. The primary cultures were incubated at 37°C with 5% CO_2_ and the culture medium was refreshed every 3–4 days. One to 3 weeks after plating, the proliferating fibroblast population could be observed near the minced tissue. The primary fibroblasts were then passed, and the remnant tissues were discarded. The expression of vimentin and α-SMA and absence of CK were used to confirm the identity of normal or CAFs. Most primary fibroblasts undergo senescence after 10–15 continuous passages, characterized by increased size and decreased proliferation rate.

For the generation of CM, fibroblasts were seeded in 24-well culture plates at 50,000 cells per well. When the cells reached 80% confluency, the culture medium was replaced with RPMI-1640 containing FBS (2% for WB-related experiments and 6% for transwell-related experiments). Three to 4 days later, the culture medium was harvested and centrifuged at 10,000 g for 5 min; the supernatant was collected as fibroblast CM for subsequent experiments.

### Lentiviral Plasmid Construction

RAB31 cDNA (Youbio, G118068, Hunan, China) was used as template, and XhoI and BamHI restriction sites were introduced into the cDNA using PCR. RAB31 was inserted to a 2nd-generation lentiviral vector containing a CMV promotor with EGFP reporter gene (pLV-EGFP-2A, VL3401, Inovogen Tech, Beijing, China). For gene knockdown, reverse complementary ssDNA oligos were annealed in M buffer (Clontech, Mountain View, CA, USA) using a gradient cooling PCR program. The resulting dsDNA was inserted into a 2nd-generation lentiviral vector containing a U6 promotor and an EGFP reporter gene (pLVshRNA-EGFP, VL3101, Inovogen Tech, Beijing, China) using a homologous recombination kit (#10911ES25, Yeason, Shanghai, China). Lentivirus packaging plasmids pSPAX2 (VT1444) and pMD2.G (VT1443) were purchased from Youbio, China. Primers used for qPCR and Oligos used for knockdown are included in Supplementary Tables 1, 2, respectively.

### Lentivirus Production and Infection

LentiX-293T (Clontech, Mountain View, CA, USA) was grown in a 100-mm culture dish with 12 ml DMEM containing 10% FBS and 100 U/ml streptomycin and penicillin. At 80% confluency, a total of 25 μg DNA including lentiviral vector, pSPAX2, and pMD2.G packaging vectors were transfected into the 293T cells using Lipofectamine 2000 transfection reagent (Thermo Fisher Scientific, Waltham, MA, USA) at a ratio of 2:2:1. The supernatant was collected at 36 h and 48 h and filtered through a 45-μm filter. The virus was concentrated using a sterilized 5× concentration reagent [8.766 g NaCl, 50 mg PEG 8000 (Sigma-Aldrich, St. Louis, MO, USA) in 200 ml Milli-Q water] which was added to the supernatant and mixed overnight and centrifuged at 4,000 g for 30 min at 4°C. The precipitated sediment was dissolved in Opti-MEM for 100× concentration.

For lentivirus infection, fibroblasts were seeded in 6-well plates at 300,000 cells per well. Eighty microliter concentrated lentivirus was added to each well and incubated overnight before refreshing the culture medium. Three days after transfection, the cells were observed under a fluorescence microscope for EGFP expression and western blotting was used for the validation of overexpression or knockdown efficiency. Stably transfected CAFs were denoted *CAF EGFP* for vector control, *CAF RAB31* for RAB31 overexpression, and CAF RAB31 KD for RAB31 knockdown. Similarly, *RKO MET KD* referred to knockdown of MET in RKO.

### Transwell Assay

Transwell assay was performed using 8-μm, 24-well plates (Corning Costar, Lowell, MA, USA). Thirty thousand cells suspended in 200 μl RPMI-1640 supplemented with 6% FBS were seeded in the upper chamber. The lower chamber contained RPMI-1640/6% FBS mixed with fibroblast-CM at a ratio of 2:1 or with cytokines at a concentration of 50 ng/ml. For HGF neutralization experiments, the anti-HGF polyclonal antibody (AF-294-NA, R&D Systems, Minneapolis, MNB, USA) was added to the mixed medium and incubated at 37°C for 1 h before adding to the lower chamber. For MET drug blockade, the inhibitors were added to the cell suspension in the upper chamber and incubated for 20 min at 37°C and to the mixed medium in the lower chamber right before the transwell was inserted. The transwell was incubated at 37°C under 5% CO_2_ for 45 or 50 h. Then, the upper chamber was fixed with 4% PFA, the upper surface of the filter was wiped with a cotton swab to remove the remaining cells, and the lower surface was stained using hematoxylin–eosin or Crystal Violet. After rinsing in water, the cells were observed under a light microscopy. The number of cells was counted in three to five random fields per transwell in which the number of cells were counted manually.

### Wound Healing Assay

Cells were seeded into a silica gel mold containing two rectangular chambers divided by a septal across the middle. The mold was placed in 6-well culture dishes with each chamber containing ~30,000 cells. The cells were allowed to settle overnight before retrieving the mold (at 0 h) to leave two groups of cells of 80% confluency separated by a gap. The cells were cultured in a complete culture medium supplemented with CAF CM for 24 h. The area of the gap at 0 and 24 h was measured using the ImageJ software. The migration speed was indicated by a migration index calculated by the following formula:

$$\begin{array}{l} migration\;index=\;\frac{\text{AREA}(0\text{h})-\text{AREA}(24\text{h})}{\text{AREA}(0\text{h})} \end{array}$$

A larger migration index indicates faster migration speed.

### Western Blot Analysis (WB)

WB was performed using conventional WB procedures. Primary antibodies were purchased from CST (Cell Signaling Technology, Danvers, MA, USA), including anti-HGF anti-phospho Met (#3133), anti-cMet (#8198), anti-Akt (#2920), anti-phospho-Akt (ser473, #4060), anti-pPDGFR (Tyr1009, #3124), and anti-phospho-Erk1/2 (Thr202/Tyr204, #4370).

Human cytokine antibody array (Abcam, Cambridge, USA, ab133998) was performed according to the manufacturer's instruction manual.

For MET activation and neutralization experiments, RKO cells were seeded in 24-well plates at a density of 40,000 cell per well in culture medium containing 10% FBS. One day before treatment, the wells were refreshed with medium containing 2% FBS. CAF CM diluted with culture medium (2% FBS) at 1:2 was added to the cells for 20 min at 37°C. Alternatively, the HGF neutralizing antibody was added to the diluted CAF CM and incubated for 1 h before treatment. The cells were lysed with a RIPA-containing phosphatase inhibitor cocktail for preparation of the WB samples.

### Animal Experiments

Female 6 to 8 week-old age-matched BalbC/nude mice were used for animal studies. The mice were kept in pathogen-free colony cages (5 per cage) in an airflow cabinet at 23°C, 12/12 h day/night cycle, and free access to food and water. The mice were anesthetized with sevoflurane using a small-animal anesthesia system (RWD Life Science, Shenzhen, China). A 6–8-mm incision was made near the upper left quadrant of the mice to expose the spleen. CAFs and luciferase-RKO mixed at a ratio of 3:10 PBS were injected to the distal end of the spleen at 130,000 cells in 50 μl PBS per mice. Three weeks later, the spleen was surgically removed under anesthesia and the mice were allowed to recover for 3 days. Then, the mice received intraperitoneal injection of luciferin and were subjected to live imaging for visualization of liver metastasis using a system (IVIS Spectrum, Perkin Elmer). Then, the mice were sacrificed and 200 μl of luciferin was injected into the portal vein. The liver was then removed and subjected to bioluminescence imaging to confirm the *in vivo* observation and to rule out peritoneal metastasis caused by leakage of the tumor suspension during inoculation.

### Quantitative Reverse-Transcription PCR (RT-qPCR)

Cells were lysed in TRIzol (Invitrogen), and total RNA was extracted according to the instruction manual. cDNA was obtained using Hifair® II 1st-Strand cDNA Synthesis SuperMix for qPCR (Yeason, Shanghai, China). Real-time PCR was performed using the 7500 Fast Real-Time PCR System (Applied Biosystems, Foster City, CA, USA) with Hieff® qPCR SYBR Green Master Mix (Low Rox Plus) (Yeason, Shanghai, China) in a two-step reaction. Analysis of the relative expression of target gene was performed by the comparative Ct value.

### Cell Proliferation Assay

CCK-8 assay was used to assess cell proliferation. Cells were seeded in 96-well plates at 3,000 cells per well in triplicates. After culturing under the designated conditions, the supernatant was replaced with 100 μl fresh culture medium containing 10% FBS and 10 μl CCK-8 reagent and incubated for 1.5 before measuring with a microplate reader at 450 nm. A blank well containing only culture medium and CCK-8 reagent was also measured as a background signal, which was subtracted from the experiment readouts.

### Statistical Analysis

All analysis was performed in GraphPad Prism 8. Results were presented as means ± SD. Statistical analysis was performed using one-way ANOVA with Bonferroni *post hoc* test or the Student's *t*-test. Survival analysis was performed using log-rank (Mantel–Cox) test. *P* < 0.05 was considered to be statistically significant.

## Results

### The Localization and Expression of RAB31 in CRC

**Figure 1:**
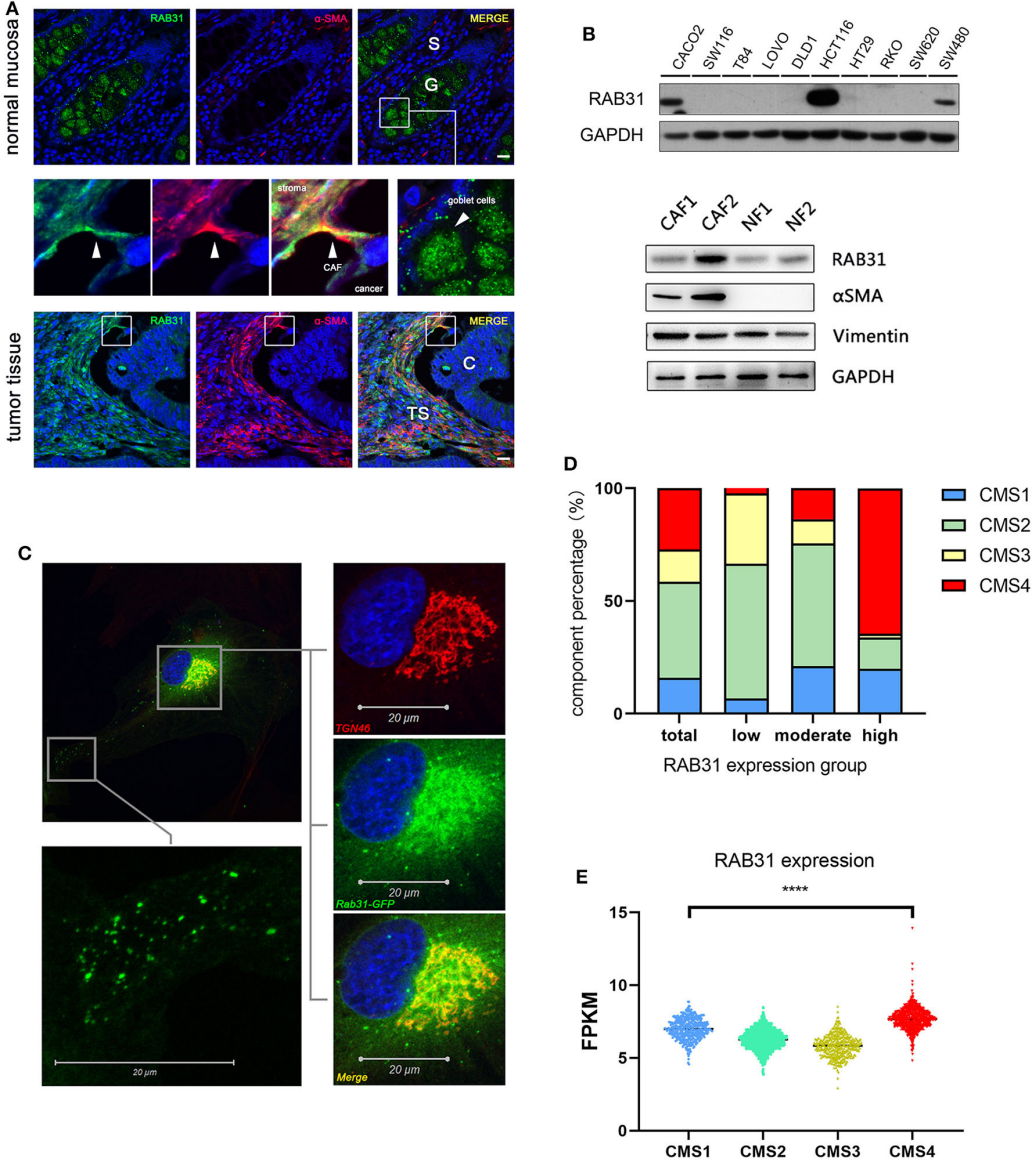
The localization and expression of RAB31 in colon cancer. **(A)** Paired colon cancer tissue and adjacent normal mucosa co-stained for RAB31 (green) and the CAF marker α-SMA (red) show significant staining of RAB31 in the goblet cells of normal mucosa which are balloon-like cells clustered together and in the tumor stroma where it partially colocalizes with α-SMA. *Scale bar* = 50 μm. S, normal stroma; C, cancer cells; TS, tumor stroma; G, goblet cells. **(B)** WB detects the expression of RAB31 in 3 out of 10 CRC cell lines and in paired primary CAFs and NF. **(C)** Subcellular localization of RAB31-EGFP shows colocalization with the trans-Golgi network marker TGN46 and cluster-like distribution in the cytoplasm of primary CAFs. **(D)** The CMS component of CRC patients in RAB31 high (*n* = 941), moderate (*n* = 941), and low expression (*n* = 962) groups with a cut-off value of 6.22 and 7.14 FPKM. The RAB31 high expression group holds the largest portion of CMS4 CRC patients. **(E)** RAB31 expression is highest in CMS4 (stromal type) colorectal cancer and significantly higher than CMS1 (immune type). Data shown as mean ± s.d. ****P < 0.0001; statistical significance was determined by one-way ANOVA with Bonferroni *post hoc* test.

Since RAB31 has never been studied in colon cancer, we validated a commercially available RAB31 antibody using WB and immunocytochemistry (Supplementary Figure 1) and explored the expression and distribution of RAB31 in paraffin-fixed tissue from colon cancer patients. Immunofluorescence staining of normal mucosa and paired tumor tissue showed that RAB31 was highly expressed in goblet cells with dispersed cluster-like distribution in the cytoplasm. The stromal compartment of normal tissue did not show significant staining of RAB31 compared to the goblet cells. In tumor tissue, RAB31 expression in the cancer cells were generally low, while high expression was detected in the tumor stroma in a subgroup of samples. Partial colocalization with α-SMA, a marker for CAFs (Figure 1A), or vimentin, a marker for stromal cells (Supplementary Figure 2), was observed, suggesting a functional role for RAB31 in CAFs. RAB31 was detected by WB in 3 out of 10 CRC cell lines, and in 2 primary CAFs and paired normal fibroblasts (Figure 1B). As RAB family proteins are generally membrane associated, their functions may be reflected by their subcellular localization. We transiently expressed a RAB31-EGFP fusion protein in primary CAFs and found that RAB31 was accumulated near the cell nucleus and colocalized strongly with the Golgi complex marker TGN46. In the cytoplasma, RAB31 was distributed as tiny clusters resembling intracellular vesicles (Figure 1C), suggesting that RAB31 may be a mediator of Golgi-endosome transportation, which is consistent with early studies (6, 7).

Next, we analyzed the expression of RAB31 in colorectal tumor samples based on the consensus molecular subtypes (CMS) classification. Normalized gene expression data (25) of 2,844 patients were sorted by RAB31 expression level and equally divided into RAB31 high, medium, and low expression groups. The RAB31 high group consisted of a significantly higher proportion of CMS4 (high stromal content) CRC samples compared to the overall CMS4 proportion (64.55% vs. 27.10%) (Figure 1D). In addition, RAB31 overall expression was highest in the CMS4 group, significantly higher than that in the CMS1 (immune related) group, which had the second highest expression of RAB31 in the 4 CMS groups (CMS4 vs. CMS1; 7.69 ± 0.75 vs. 6.99 ± 0.70; *P* < 0.0001) (Figure 1E).

These results suggest that RAB31 may play a functional role in cellular components of the tumor stroma such as CAFs.

### Stromal Expression of RAB31 Is Associated With Poor Prognosis in CRC

**Figure 2:**
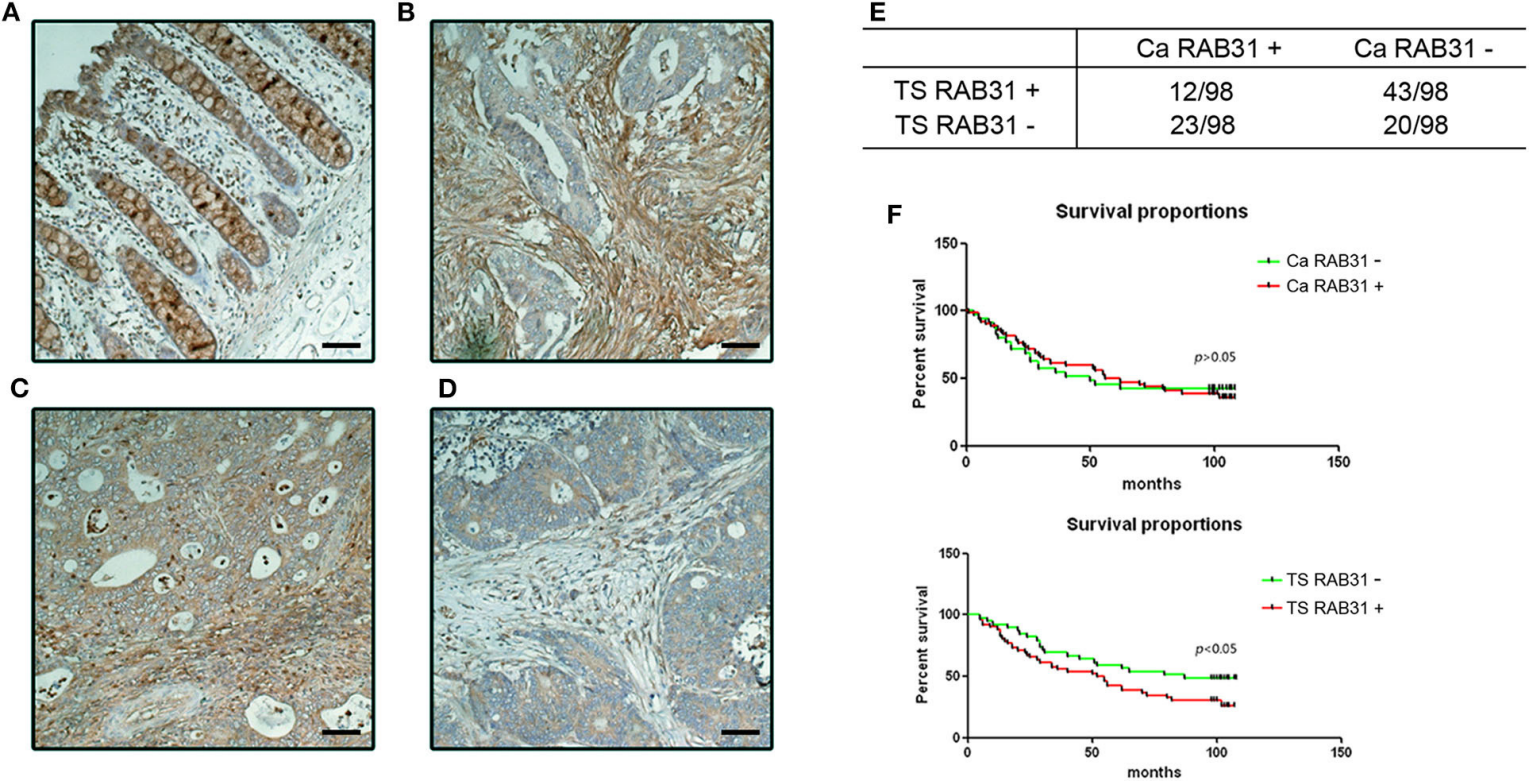
Stromal expression of RAB31 is associated with poor prognosis in CRC. RAB31 was stained in 98 colon cancer tissue and paired normal mucosa. RAB31 was found in goblet cells in normal tissue **(A)** and may be richly expressed in stroma or cancer cells **(B,C)**, or nearly absent in both compartments in some samples **(D)**. *Scale bar* = 50 μm. **(E)** RAB31 was evaluated in the cancer cells and stromal compartments for each sample. Summarized data of IHC results shows the fraction of RAB31-positive/negative samples out of 98 patients in terms of tumor stroma or in cancer cell expression. TS RAB31: tumor stromal RAB31 expression, Ca RAB31: cancer cell RAB31 expression. **(F)** Kaplan–Meier survival analysis of RAB31 expression based on stromal or cancer cell expression suggests that only stromal RAB31 expression was significantly associated with survival, *P* = 0.018, by log-rank (Mantel–Cox) test.

To further investigate the clinical significance of RAB31 expression in colon cancer, we performed immunohistological staining of RAB31 in 98 CRC tumor samples (Table 1). The expression of RAB31 was graded in both the cancer cells and tumor stromal compartment using a 3-tier scale: 1, no staining or weak staining; 2, moderate staining; 3, strong staining. The results were then divided into negative staining for grade 1; positive staining for grade 2 and 3. While RAB31 may be expressed in both cancer cells and stromal cells (Figures 2A–D), a higher percentage of stromal RAB31 expression was observed (56.12% vs. 35.71%) (Figure 2F). Kaplan–Meier survival analysis was performed on RAB31-positive vs. negative groups in cancer cells and stromal compartments, respectively. We found that CRC with stromal expression of RAB31 was associated with poor survival in comparison to those with negative staining. Interestingly, expression of RAB31 in cancer cells was not correlated with survival (Figure 2E).

**Table 1:** Basic characteristics of CRC patients.

Mean age (SD)		68.9 ± 11.8
Sex (%)	Male	52 (53.1%)
	Female	46 (46.9%)
TNM stage		
	I	6 (6.1%)
	II	53 (54.1%)
	III	35 (35.7%)
	IV	4 (4.1%)
Site		
	Right colon	49
	Left colon	48
	N/A	1

### CAF Expressed RAB31 Promotes CRC Cell Migration and Metastasis

**Figure 3:**
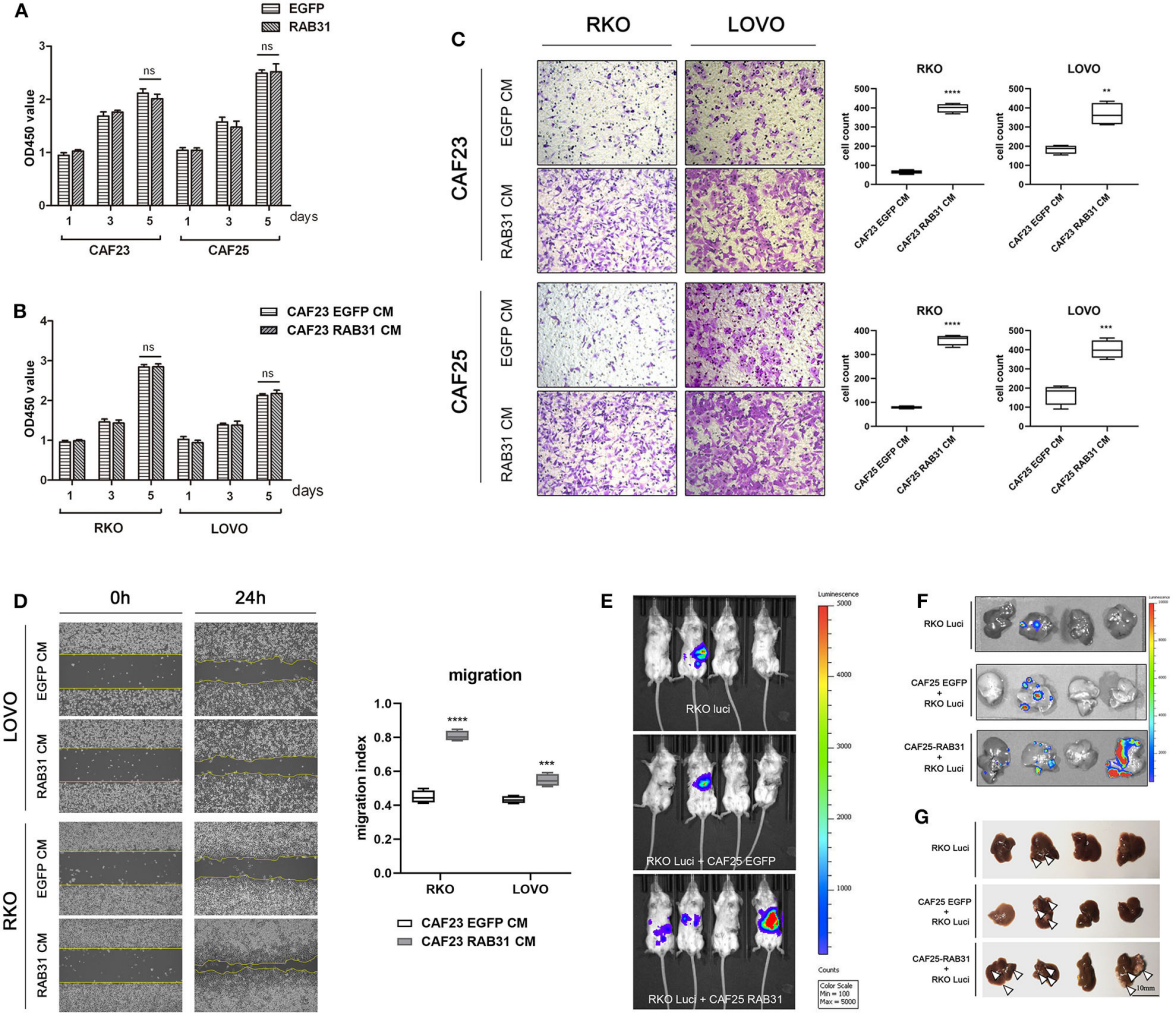
CAF-expressed RAB31 promote colorectal cancer cell migration and metastasis. **(A)** The proliferation rate of CAF23 and CAF25 overexpressing RAB31 was unchanged compared to vector control CAFs measured by cck-8 assay. **(B)** Proliferation of RKO and LOVO cells incubated with conditioned medium obtained from RAB31-overexpressing CAFs or vector control CAFs measured by CCK-8 assay. **(C)** Conditioned medium of CAFs overexpressing RAB31 significantly facilitated the migration of CRC cell lines RKO and LOVO compared to vector control measured by Transwell assay at 45 h. **(D)** Wound healing assay of RKO and LOVO treated with CM from RAB31-overexpressing CAFs or vector control CAFs for 24 h. **(E)** RKO colon cancer cells expressing luciferase inoculated into the distant end of the spleen in mixture with RAB31-overexpressed CAFs exhibited increased liver metastasis compared to that mixed with or without WT CAFs. Images of livers with metastatic lesions at 30 days detected by live imaging. **(F)** Bioluminescence image of metastatic lesions in isolated livers and **(G)** by direct observation, indicated by white arrowheads. **(A–D)** Data shown as mean ± s.d. ****P < 0.0001, ***P < 0.001, **P < 0.01; statistical significance was determined by Student's *t*-test.

To determine whether elevated expression of RAB31 in CAFs can affect the biological properties of CRC cells, we overexpressed RAB31 in two primary colon cancer-derived CAFs using lentiviral infection. The proliferation rates of the CAFs were unaltered following RAB31 overexpression (Figure 3A). CM obtained from these CAFs had no observable effect on cancer cell proliferation (Figure 3B) but stimulated the transwell migration of RKO and LOVO cells significantly in comparison to vector control (*P* < 0.0001) (Figure 3C), suggesting an increase in CAF-secreted migration-promoting factors. This phenomenon was further supported by wound healing assay (Figure 3D). Interestingly, while the fibroblast CM induced morphological changes in certain cell lines, the proliferation of RKO and LOVO was unaltered. The percentage of FBS supplement or the addition of fibroblast growth factor seemed to have the most prominent effect in facilitating RKO and LOVO proliferation (Supplementary Figure 3).

To further confirm our observation *in vivo*, we used a murine xenograft model for CRC liver metastasis by spleen injection of luciferase-expressing RKO. Results showed that mixed inoculation of RAB31-overexpressing CAFs and RKO exhibited a higher liver metastasis rate compared to the RKO/control CAF group or RKO alone (75, 41.67, and 25%, respectively) (Figure 3E, Table 2 and Supplementary Figure 4). After live imaging, the livers were excised for organ luminescence detection and direct observation of metastatic lesions, which was consistent with the live imaging results (Figures 3F,G).

**Table 2:** Mice liver metastasis model.

**Group**	**Total no. of mice**	**Liver metastasis**	**Rate (%)**
RKO-luciferase	8	2	25.00
RKO-luciferase+CAF25 EGFP	12	5	41.67
RKO-luciferase+CAF25 RAB31	12	9	75.00

These results suggest that CAF-expressed RAB31 may promote the migration of CRC cells through paracrine actions. Moreover, our murine xenograft model supports the clinical observation that higher RAB31 expression in the stroma is associated with CRC liver metastasis (Stage IV).

### CAF Expressed RAB31 Promotes CRC Cell Migration Through Regulating HGF Secretion

**Figure 4:**
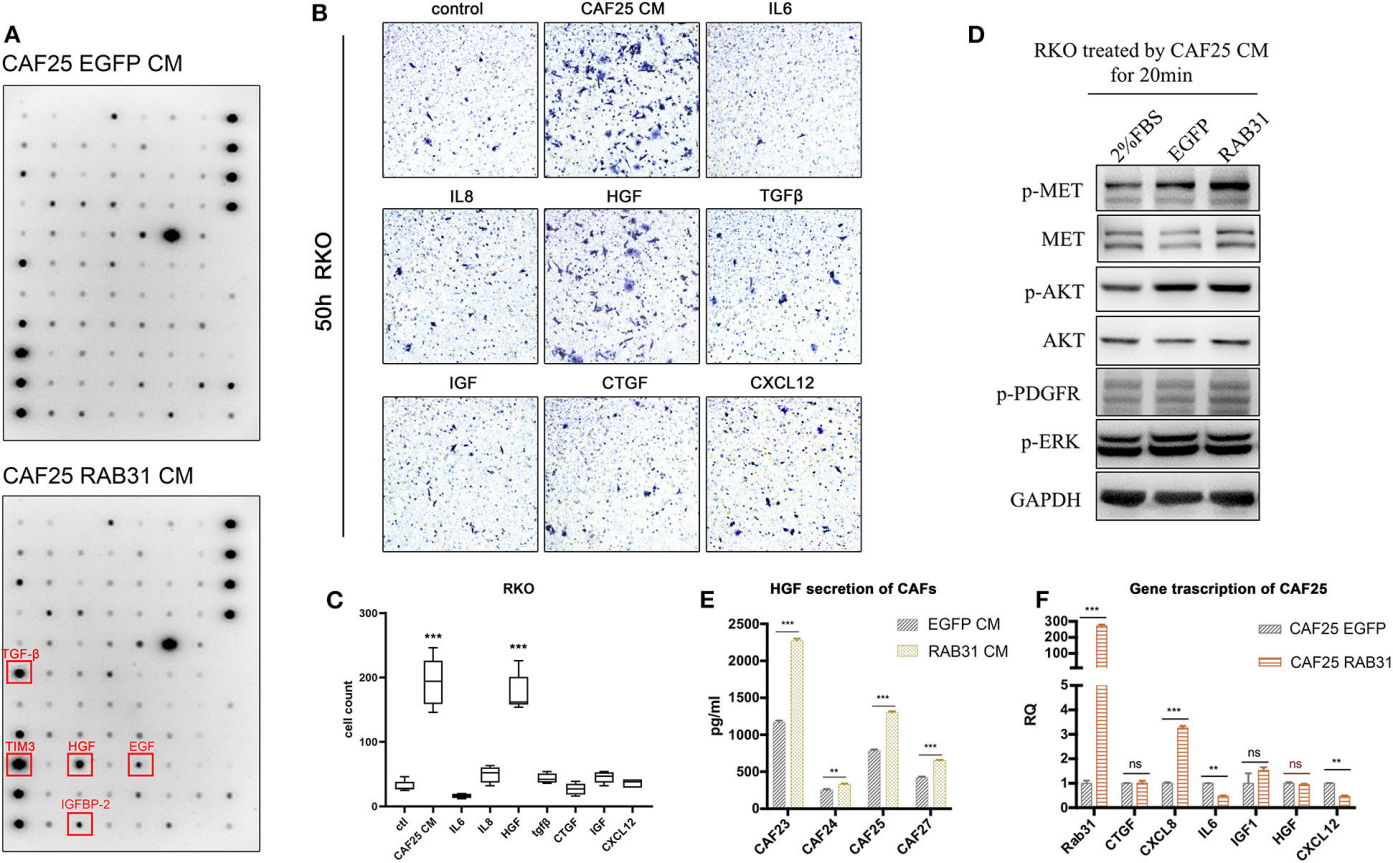
CAF expressed RAB31 promoting CRC migration by regulating HGF secretion. **(A)** Cytokine antibody array of CM from RAB31-overexpressing CAFs and vector control CAFs; red frames indicate upregulated cytokines. **(B)** Cytokines and growth factors commonly expressed by CAFs were tested for their effect in promoting RKO migration using transwell assay. **(C)** CAF CM and HGF (50 ng/ml) significantly promoted the migration of RKO in transwell assay. Data shown as mean ± s.d. ***P < 0.001 vs. control; statistical significance was determined by one-way ANOVA with Bonferroni *post hoc* test. **(D)** Detection of MET phosphorylation in RKO cells receiving 20 min treatment of CM from RAB31-overexpressing CAFs or vector control CAFs. **(E)** Comparison of HGF protein level in conditioned medium from RAB31-overexpressing and vector control CAFs by ELISA. Data shown as mean ± s.d. ***P < 0.0001, **P < 0.001 vs. control; By Student's *t*-test. **(F)** mRNA level of HGF and other CAF-secreted proteins from CAFs extracted total mRNA measured by QPCR. Data shown as mean ± s.d. ***P < 0.0001, **P < 0.001 vs. EGFP CAF (vector control) by Student's *t*-test.

To further identify the growth factors or cytokines responsible for the enhanced migration-inducing ability of CM from RAB31-overexpressing CAFs, we used a cytokine antibody array panel to search for differentially secreted factors. Quantification of the immune blots revealed TGF-β, HGF, EGF, and IGFBP-2 and TIM3 (Figure 4A) to be significantly elevated in the CM of RAB31-overexpressing CAFs. Expression changes of other secreted factors can be found in Supplementary Figure 7. CAFs have been known to secrete a myriad of cytokines and growth factors such as SDF-1, IL-6, IL-8, TGF-β, and HGF (31, 32). While many studies claim that these CAF-secreted factors may enhance tumor cell migration and induce epithelial–mesenchymal transition (EMT), they may play distinct roles in different types of cancers. Transwell assay results showed that CAF-derived CM potently promoted RKO migration. In addition, among 7 well-studied CAF-secreted factors, HGF exhibited the strongest effect in promoting RKO migration (*P* < 0.0001) (Figures 4B,C). The combined results of cytokine array and transwell assay suggest that HGF may be a key factor in CAF-mediated migration of colon cancer cells.

Next, we treated RKO cells with CM from control or RAB31-overexpressing CAFs and examined the activity of the HGF/Met signaling pathway. Results showed that incubation with control CAF-derived CM increased MET phosphorylation in RKO; this effect was further enhanced when incubated with that from RAB31-overexpressing CAFs (Figure 4D). HGF protein level was significantly increased in the CM of four different primary RAB31-overexpressing CAFs compared to their vector control counterparts by ELISA detection (Figure 4E), while the mRNA level of HGF was unchanged (Figure 4F).

These results suggest that RAB31 may mediate posttranslational regulation of HGF expression in CAFs, which subsequently increase HGF/MET signaling in cancer cells.

### HGF/Met Signaling Blockade Inhibited CRC Cell Migration Mediated by RAB31 Expression in CAFs

**Figure 5:**
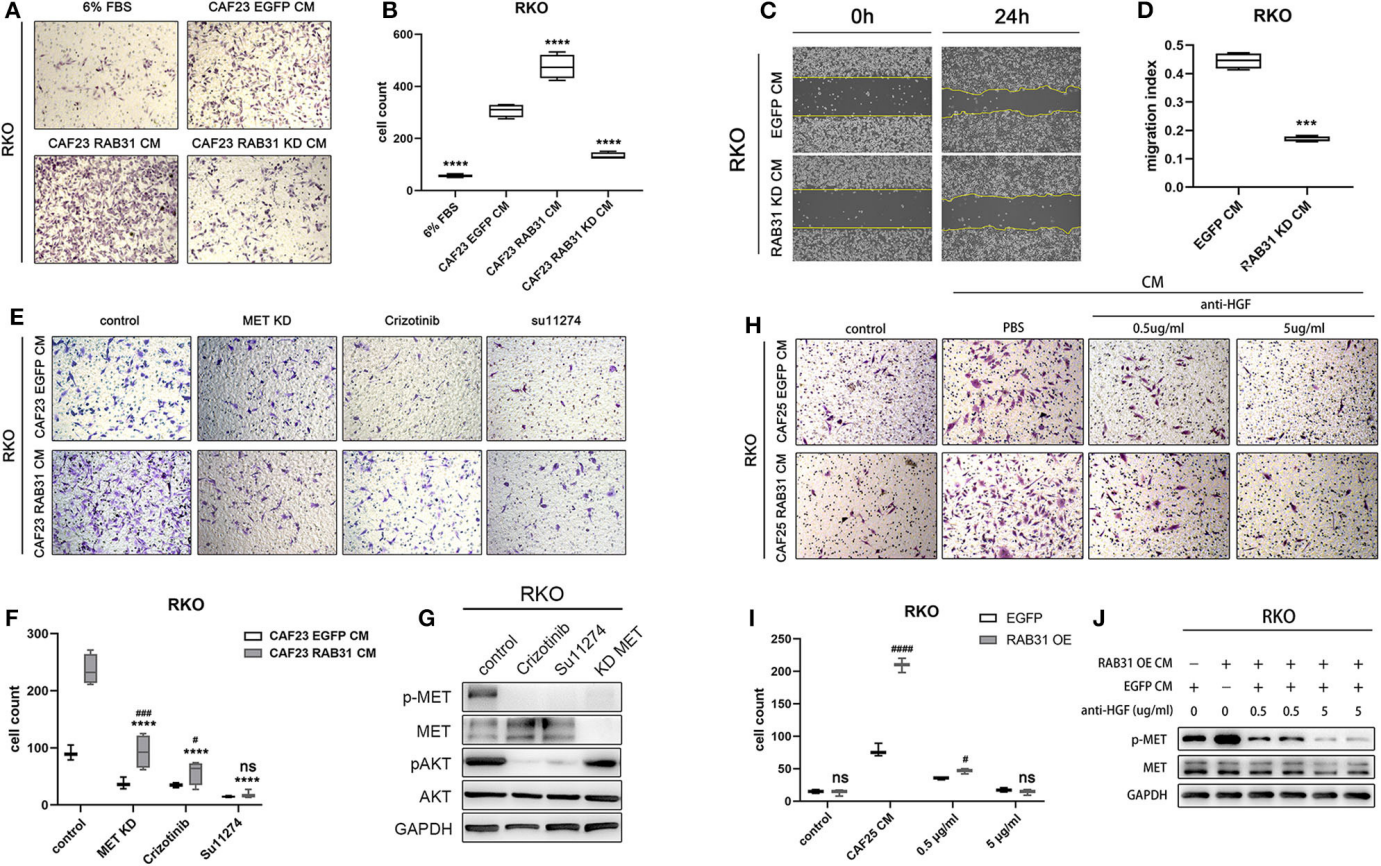
HGF/MET signaling blockade abolishes colon cancer cell migration mediated by RAB31 in CAFs. **(A)** RKO migration measured by Transwell assay at 50 h. The lower chamber contained primary culture medium supplemented with 6% FBS for control or CAF derived CM. **(B)** The migration of RKO was significantly enhanced by vector control (EGFP) CAF CM, and further enhanced by RAB31 overexpressing CAF CM measured by Tranwell assay. Knockdown of RAB31 in CAF by lentivirus significantly reduced the migration of RKO. ****P < 0.0001 vs. CAF23 EGFP CM. **(C,D)** Wound healing assay of RKO cells incubated with CM from vector control CAFs or RAB31 knockdown CAFs. ***P < 0.001 vs. RAB31 KD CM. **(E)** Migration of RKO cells treated with RAB31 overexpressing CAF CM or EGFP CAF CM (vector control) in the presence of MET inhibitors Crizotinib (100 nM) or Su11274 (5 μM) measured by Transwell assay at 45 h. **(F)** Blockade of MET signaling significantly inhibited the CAF CM mediated RKO migration and ameliorated the promigratory effect of RAB31 overexpression. ^****^P < 0.0001 vs. CAF23 RAB31 CM control; ^*###*^*P* < 0.001, ^#^*P* < 0.05 vs. CAF23 EGFP CM. **(G)** WB indicated that treatment of RKO with CAF23 EGFP CM increased the phosphorylation of MET, which was blocked by MET inhibitors or by MET knockdown in RKO. **(H,I)** Neutralizing HGF in CAF25 EGFP CM and CAF25 RAB31 CM inhibited migration of RKO cells by Transwell assay at 45 h in a dose dependant manner and abolished the difference of the two CMs at 5 μg/ml. ^*####*^*P* < 0.0001, ^#^*P* < 0.05 vs. CAF23 EGFP CM. **(J)** WB results showed that the increased MET phosphorylation in RKO induced by CM from RAB31 overexpressing CAFs was diminished upon preincubation with anti-HGF antibody at 5 μg/ml. **(D,F,G,I)** Data shown as mean ± s.d. Statistical significance was determined by Student's *t*-test.

Finally, we knocked down RAB31 in CAF23 significantly using lentiviral infection (Supplementary Figure 5) and found that the increase in RKO migration induced by CAF CM was partially abolished as measured by transwell assay (Figures 5A,B). In addition, wound healing assay also showed that the downregulation of RAB31 hampered CAF CM induced migration (Figures 5C,D). To further demonstrate that the HGF/Met pathway was involved, we compared the effect of CM from RAB31-overexpressing CAFs and vector control CAFs on RKO migration in the presence of the small molecule MET inhibitors crizotinib or Su11274, or by knockdown of MET in RKO. Results showed that knockdown of MET in RKO or treatment with crizotinib (100 nM) or Su11274 (5 μM), respectively significantly reduced the migration of RKO induced by CAF-derived CM. While RAB31-overexpressing CAF-derived CM increased RKO migration to a greater extent compared to control CAFs, this difference was diminished in the MET knockdown and crizotinib groups and completely abolished in the Su11274 group (Figures 5E,F). The inhibitory effects of the two inhibitors and MET knockdown were validated by WB which showed significantly decreased phosphorylation of MET and downstream Akt signaling upon inhibitor preincubation (Figure 5G). Because there are potentially other growth factors that can activate MET such as IGF (33), we further complemented our study by neutralizing HGF in CAF CM. The HGF-neutralizing antibody reaches a maximum effect at 5 μg/ml as indicated by significantly decreased p-MET (Supplementary Figure 8). Transwell assay showed that neutralizing HGF inhibited migration of RKO and abolished the pro-migratory difference between CAF25 RAB31 CM and CAF25 EGFP CM (Figures 5H,I). In addition, 5 μg/ml neutralizing antibody reduced CM-induced p-MET of the two CAFs to similar levels (Figure 5J), suggesting HGF as the primary growth factor responsible for CM induce MET activation.

Taken together, these results strongly suggest that RAB31 overexpression in CAFs may promote RKO migration through paracrine regulation of HGF/MET signaling.

## Discussion

In our previous study, RAB31 expression was found to be significantly elevated in stage IV CRC compared to normal tissue and stage I–III CRC (2). In this study, RAB31 was further identified to be highly expressed in CMS4 or stromal type CRC. Stromal RAB31 expression had worse prognosis in colon cancer. We demonstrate that increased RAB31 expression in CAFs, a major component of stromal cells, may promote colon cancer progression by increasing HGF secretion which subsequently activates HGF/Met signaling in CRC cells.

While studies have investigated the role of RAB31 in the cancer cells of hepatic cancer, breast cancer, pancreatic cancer, gastric cancer, and glioblastoma (10–12, 34, 35), we described the functional role of RAB31 in colon cancer-derived CAFs because its expression in the tumor stroma but not cancer cells predicted poor survival. RAB31 was expressed in goblet cells of normal colon mucosa as well as in cancer cells in a fraction of colon cancer samples. Its expression in cancer cells may be a premature feature of goblet cells since cancer cells are genetically unstable and incompletely differentiated (36). On the other hand, CAFs are genetically stable but heterogeneous in origin (37, 38). Functional CAF subpopulations have been classified based on expression of specific marker proteins (39, 40). Our results show that not all CAFs express RAB31 in paraffin-fixed tissue by IHC. In addition, WB results showed that cultured CAFs did not show a significantly higher expression of RAB31 compared to their NF counterpart. Therefore, increased RAB31 expression in the tumor stromal may be induced by specific factors in the microenvironment or may mark a specific subtype of CAFs. This requires further investigation under the basis of an authentic subgroup classification system of CAFs, which has yet to be established.

CAFs secrete a myriad of proteins such as SDF1 (CXCL12), TGF-β, IGF, and CTGF, which have been shown to induce EMT (40–43), increasing the motility of cancer cells to promote metastasis. Using cytokine antibody array, we found several cytokines and growth factors upregulated in the CM of RAB31-overexpressing CAFs. Among them, HGF seemed to have the most pronounced effect in promoting the migration of CRC cell lines RKO and LOVO. HGF is a fibroblast-derived growth factor found to stimulate the mobility of epithelial cells (44–46) and was later shown to play an important role in the progression of malignant tumors (26). HGF is the canonical ligand for the receptor tyrosine kinase MET. However, it has been demonstrated that MET can be activated through HGF-independent mechanisms such as gene amplification, mutation, and transcriptional upregulation (47–49). Moreover, interaction with other proteins such as Integrin α6β4, intercellular adhesion molecule 1 (ICAM-1), and CD44v6 enhances MET activation (50, 51). Transactivation of MET by GPCRs and EGFR which bind to multiple ligands has also been reported (52). In our study, we found that treatment with CAF-derived CM promoted MET activation, which was further enhanced using CM from RAB31-overexpressing CAFs. Moreover, depletion of HGF using a polyclonal antibody reduced RKO MET activation by CM from RAB31-overexpressing CAFs and control CAFs to similar levels, suggesting that enhanced MET activation by RAB31-overexpressing CAF CM was indeed mediated primarily by HGF and not by other soluble factors.

Approximately 70 RAB proteins have been identified in the human genome. The expression of RAB family proteins is dysregulated in a variety of tumor types, which may alter the biological properties of cancer cells such as proliferation, migration, and invasion (9). RAB proteins switch between a GTP bound “on state” and GDP bound “off state,” allowing them to bind effector proteins and determining localization on membrane compartments or the cytoplasm (53). RABs that reside in the Golgi complex are implicated in maintaining the Golgi structure, Golgi to endosome trafficking, and protein sorting. Although we described the subcellular distribution of RAB31 as vesicle-like clusters, we were not able to verify whether these were anterograde-transporting vesicles. The RAB31 clusters did not colocalize with early endosomes or lysosomes (Supplementary Figure 6), which exclude its role in the endosome/lysosome degradation pathway and, to some extent, suggest that it may participate in anterograde trafficking or secretion of certain proteins.

RAB31 overexpression in CAFs resulted in increased HGF protein levels in the conditioned medium while the mRNA levels were unchanged. This further indicates that the regulation of HGF occurs at the posttranslational level. Other RAB family proteins such as RAB3C have been reported to regulate the secretion of IL6 to promote colorectal cancer metastasis, the underlying mechanism of which still needs to be elucidated (54). Different RABs may play distinct roles in the secretary pathway. The Golgi RAB8 and RAB26 interact with adrenergic receptors through different binding domains, respectively, to regulate the proper membrane expression of these GPCRs (55). RAB27 directly interacts with myosin-Va within a functional complex to facilitate the transport of dense core secretory granules to the plasma membrane in hormone and neuropeptide-producing cells or mast cells (56, 57). RAB31 has been reported to directly interact with the adapter protein APPL2, which is required for FcR-mediated PI3K/Akt signaling in macrophages (58). RAB31 has also been shown to co-immunoprecipitate with the p75 neurotrophin receptor (59) and with EGFR (60), playing an important role in the trafficking of the respective receptors. The specific binding domain or motif responsible for interaction with other proteins has never been identified in RAB31. Nevertheless, it is still possible that RAB31 may bind to HGF directly to facilitate its anterograde trafficking and secretion or serve as a functional component of a transportation complex that promotes HGF secretion.

Our study mainly focused on RAB31 in CAFs; however, the tumor stroma consists of a variety of cells including macrophages, lymphocytes, and epithelial cells. It is possible that RAB31 expressed in other cell populations may also affect the biological properties of CRC tumor cells. Therefore, it would be interesting to investigate the functional role of RAB31 in other cell types such as immune cells since CMS1 (immune infiltration type) colorectal cancer also express high levels of RAB31.

In conclusion, we present RAB31 as an important regulator of HGF secretion in CRC-derived cancer-associated fibroblasts, which plays an important role in the progression of colon cancer through activating HGF/Met signaling in cancer cells.

## Data Availability Statement

The raw data supporting the conclusions of this article will be made available by the authors, without undue reservation.

## Ethics Statement

This project was approved by the Ethical Committee of the Second Affiliated Hospital of Zhejiang University School of Medicine and written informed consent was obtained from all patients.

## Author Contributions

TY and WZ contributed equally to this study, performed the experiments, analyzed the data, and wrote the manuscript. WZ designed the experiments. TY, XQ, and LY funded the project. ZS and DK checked and revised the manuscript and confirmed all the data in the manuscript. GX and WJ performed the experiments for the revised manuscript. All authors read and approved the final manuscript.

## Conflict of Interest

The authors declare that the research was conducted in the absence of any commercial or financial relationships that could be construed as a potential conflict of interest.
