# Supplementary material for: Increased RAB31 Expression in Cancer-Associated Fibroblasts Promotes Colon Cancer Progression Through HGF-MET Signaling
Source: Front Oncol. 2020 Sep 23;10:1747. doi: 10.3389/fonc.2020.01747 (PMC7538782; doi:10.3389/fonc.2020.01747)
Supplement: Supplementary file 1 [file Data_Sheet_1.docx]

Supplementary Material

**
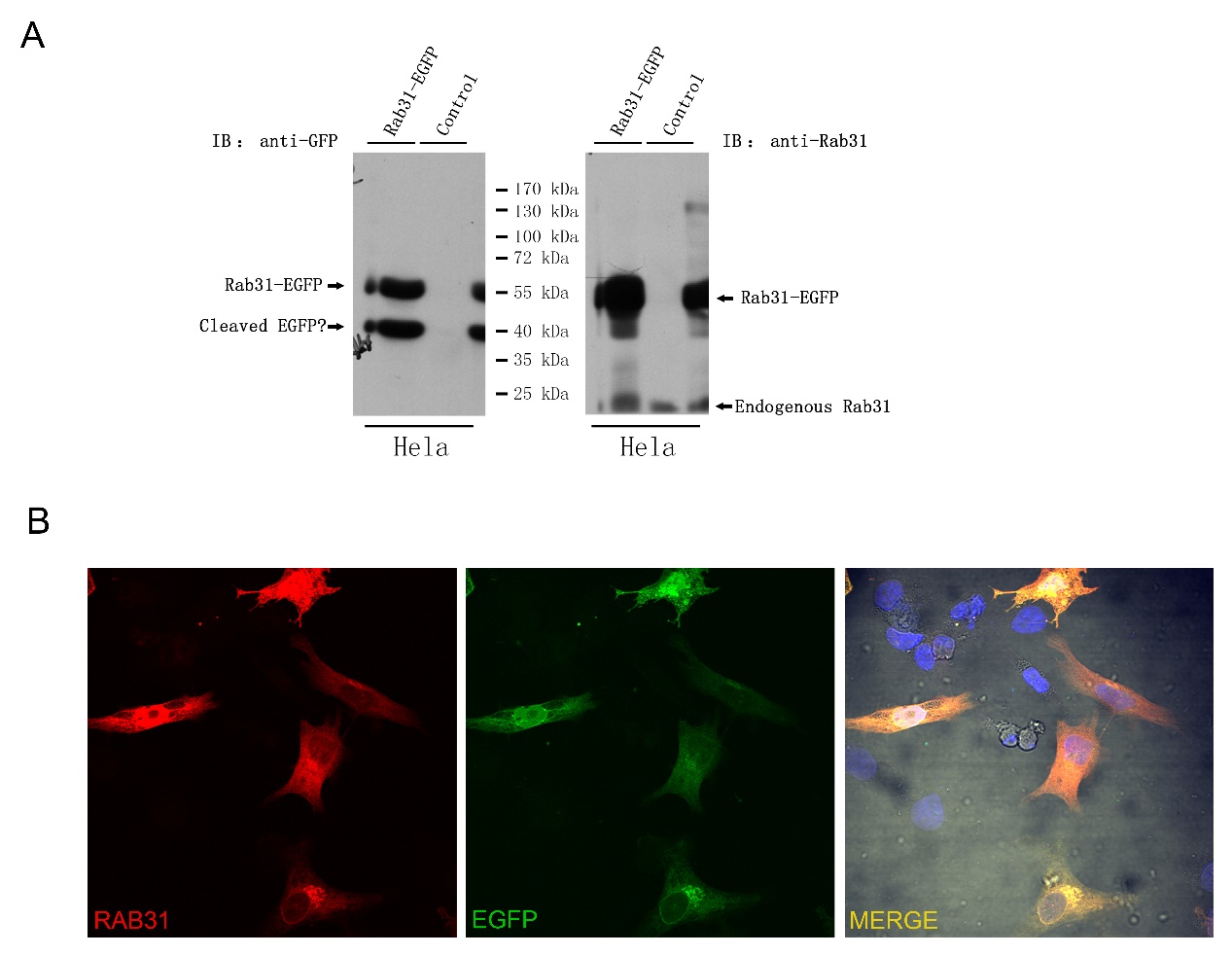
**

Supplementary figure 1.

Validation of anti-RAB31 antibody. (A) Hela cells transiently transfected with RAB31-EGFP fusion protein. WB detection of EGFP and RAB31 showed increased expression of RAB31-EGFP, while native RAB31 remained comparable in the two groups. (B) Immunofluorescence staining of RAB31 in RAB31-EGFP transfected Hela cells.

**
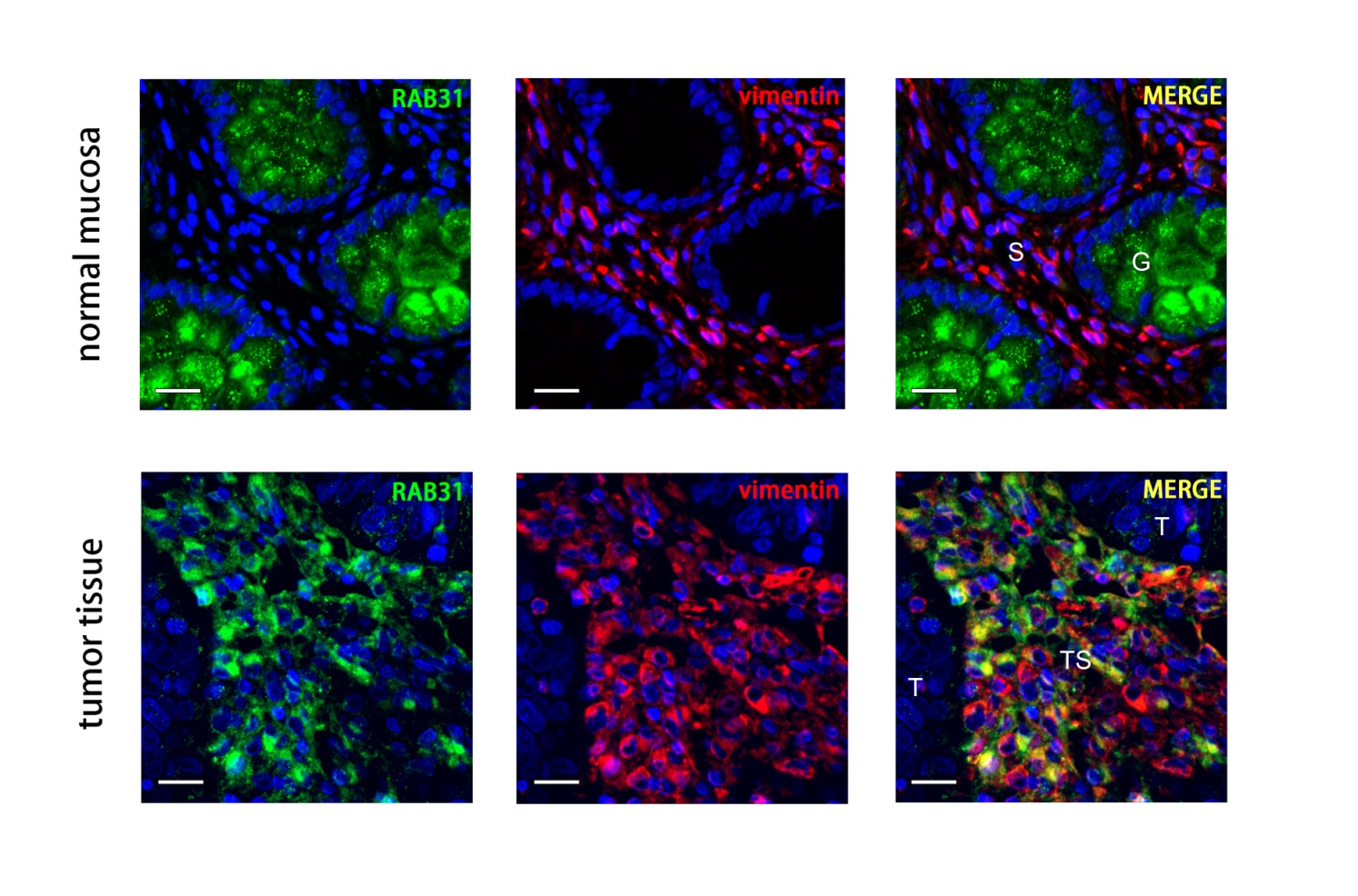
**

Supplementary figure 2.

Co-staining of RAB31 and vimentin in colon cancer and paired normal tissue. RAB31 was detected in goblet cells and the tumor stroma, and was generally absent in tumor cells. Vimentin was detected in the stroma of both normal mucosa and tumor tissue. S: normal mucosa stroma, G: goblet cells, T: tumor cells, TS: tumor stroma.

**
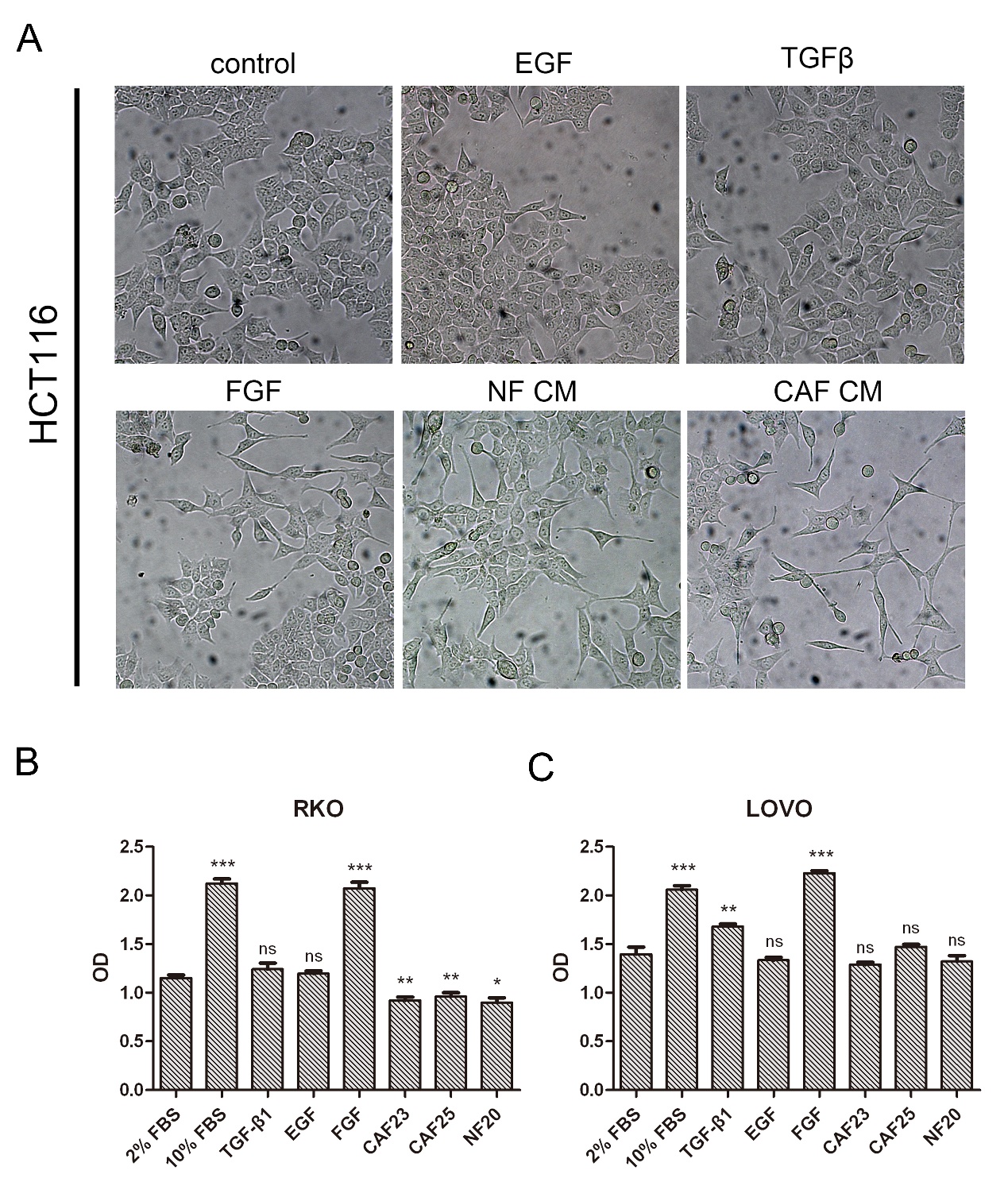
**

Supplementary figure 3.

Effect of cancer associated fibroblast conditioned medium on colorectal cancer cell lines. (A) The colon cancer cell line HCT116 was treated with PBS, EGF, TGFβ, FGF, normal fibroblast conditioned medium or cancer associated fibroblast conditioned medium for 48 hours. The two fibroblast conditioned media and FGF induced morphological changes in HCT116 featuring increased protrusions and narrower bodies. (B and C) Proliferation of RKO and LOVO cells treated with different growth factors or fibroblast derived conditioned medium for 5 days measured by CCK-8 assay . Data shown as mean ± s.d. **P*<0.05, ***P*<0.01, ****P*<0.001, compared to 2% FBS; Statistical significance was determined by One-way ANOVA with Bonferroni post hoc test.


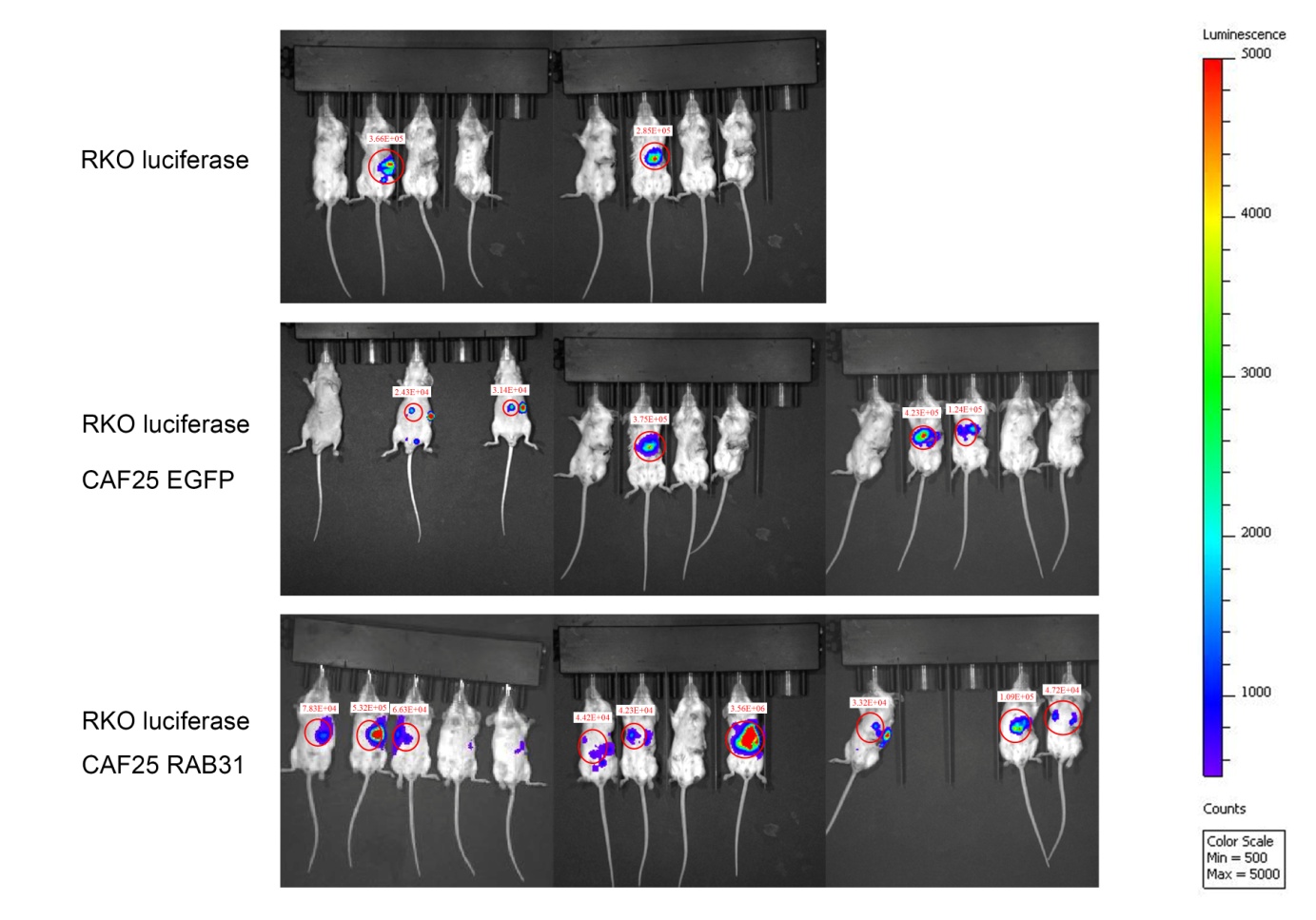


Supplementary figure 4.

Imaging and quantification of bioluminescence of liver metastasis.

**
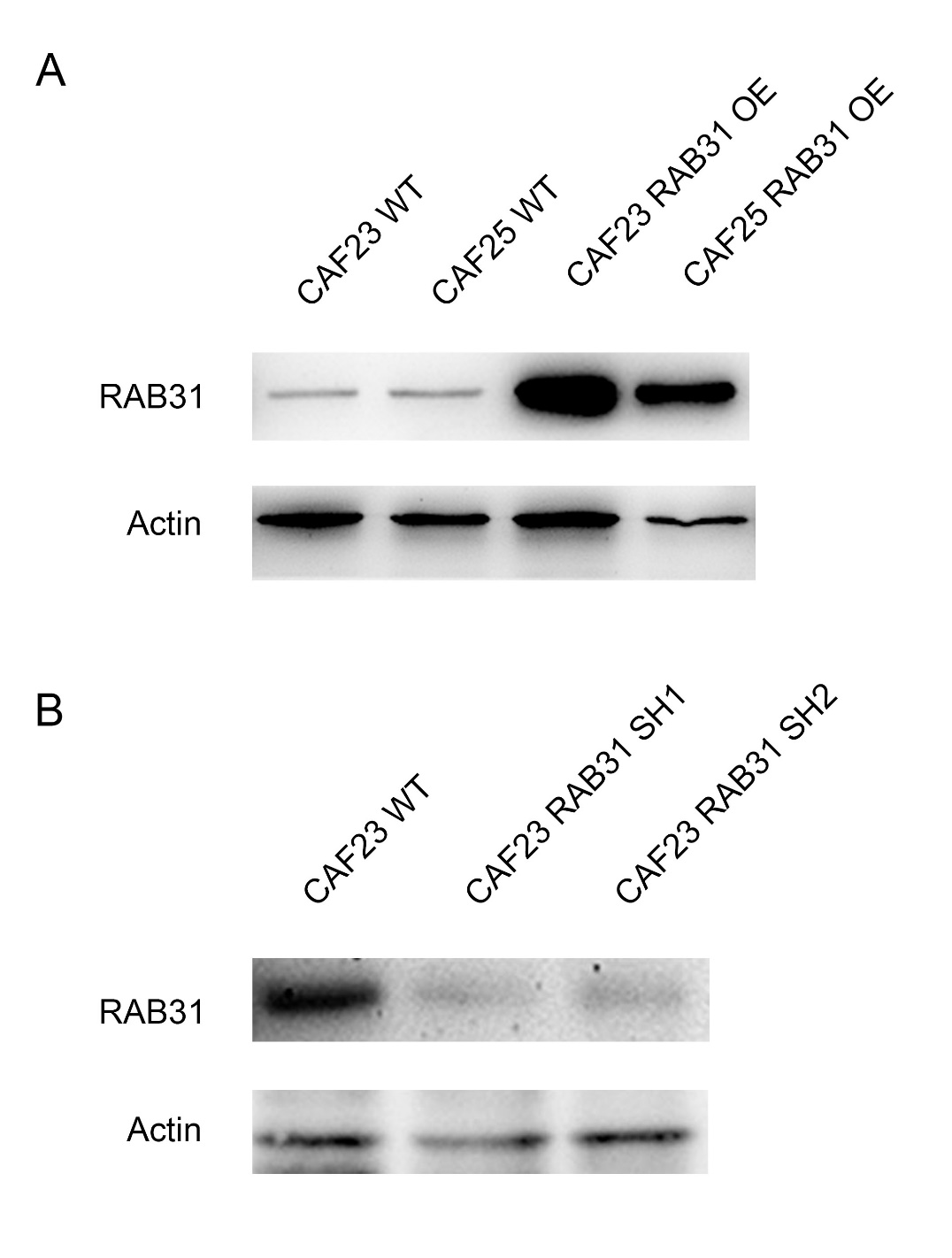
**

Supplementary figure 5.

WB detection of RAB31 overexpression and knockdown in CAFs. (A) Stably expressed RAB31 in CAF23 and CAF25. (B) Stable knockdown of RAB31 in CAF23 by lentiviral transfection. SH1 and SH2 are two sh-lentivirus plasmid clones expressing the same shRNA sequence which were validated during plasmid construction.

**
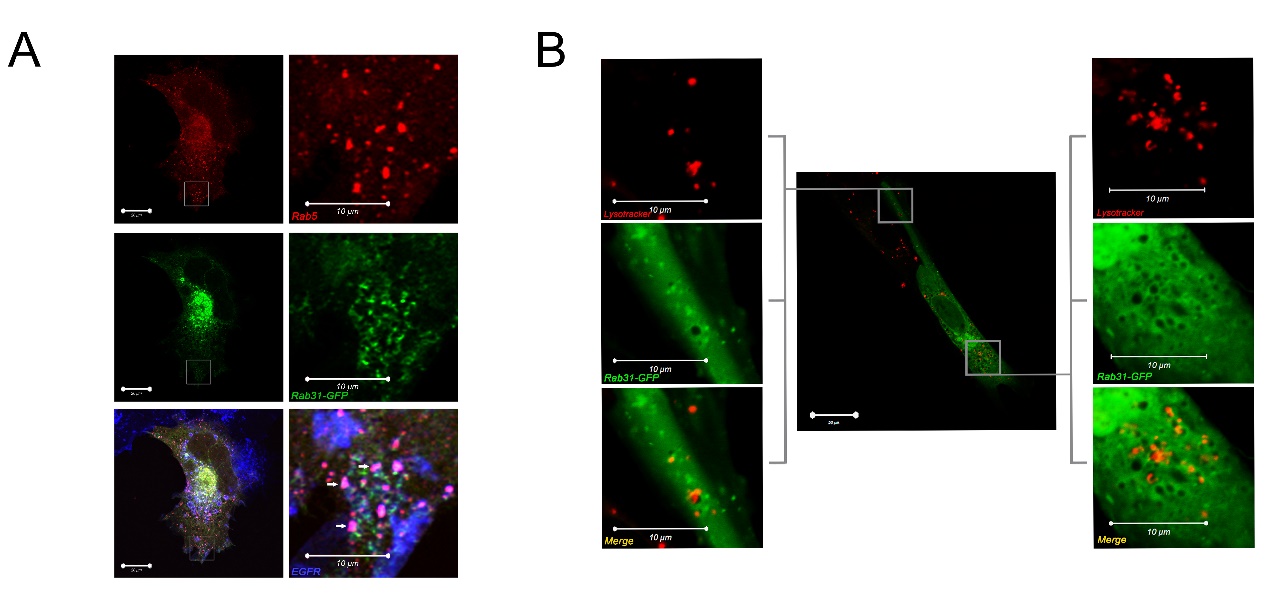
**

Supplementary figure 6.

Subcellular colocalization of RAB31. (A) Primary CAFs transfected with RAB31-EGFP and RAB5-RFP, a RAB protein enriched in early endosomes, was treated with 20 ng/ml EGF treatment for 15 min. This treatment results in the internalization of EGFR into early endosomes, a common feature of receptor tyrosine kinases. Confocal imaging showed colocalization of immunofluorescence stained EGFR (blue) and RAB5 (red) which are purple clusters indicated by white arrows. No significant colocalization was observed between RAB31 and RAB5 or EGFR, suggesting that RAB31 is not enriched in early endosomes (B) Primary CAFs transfected with RAB31-EGFP and stained for lysosomes by lysotracker. Peripheral cytoplasmic RAB31 clusters did not colocalize with lysosomes.

| **Pos** | **Pos** | **Pos** | **Pos** | **Neg** | **Neg** | **ENA-78** | **GCSF** | **GM-CSF** | **RO** | **GRO-α** |
| --- | --- | --- | --- | --- | --- | --- | --- | --- | --- | --- |
| I-309 | IL-1α | IL-1β | IL-2 | IL-3 | IL-4 | IL-5 | IL-6 | IL-7 | IL-8 | IL-10 |
| IL-12 p40/p70 | IL-13 | IL-15 | IFN-γ | MCP-1 | MCP-2 | MCP-3 | MCSF | MDC | MIG | MIP-1b |
| MIP-1δ | RANTES | SCF | SDF-1 | TARC | TGF-β1 | TNF-α | TNF-β | EGF | IGF-1 | Angioge-nin |
| Oncost-atin M | Throm-bopoietin | VEGF | PDGF-BB | Leptin | BDNF | BLC | Ck β 8-1 | Eotaxin | Eotaxin-2 | Eotaxin-3 |
| FGF-4 | FGF-6 | FGF-7 | FGF-9 | Flt-3 Ligand | Fractalk-ine | GCP-2 | GDNF | HGF | IGFBP-1 | IGFBP-2 |
| IGFBP-3 | IGFBP-4 | IL-16 | IP-10 | LIF | LIGHT | MCP-4 | MIF | MIP-3α | NAP-2 | NT-3 |
| NT-4 | Osteop-ontin | Osteopr-otegerin | PARC | PIGF | TGF-β2 | TGF-β3 | TIMP-1 | TIMP-2 | Pos | Pos |

| 1.48 | 1.19 | 1.02 | 0.97 | / | / | 0.73 | 0.72 | 1.02 | 0.57 | 0.28 |
| --- | --- | --- | --- | --- | --- | --- | --- | --- | --- | --- |
| 1.45 | 0.74 | 0.75 | 0.45 | 0.76 | 0.28 | 0.99 | 0.03 | 0.08 | 0.51 | 1.06 |
| 1.41 | 2.21 | 0.97 | 0.91 | 0.89 | 0.72 | 0.37 | 0.94 | 0.69 | 1.02 | 0.88 |
| 0.91 | 0.94 | 1.26 | 0.89 | 0.95 | 0.60 | 1.32 | 1.14 | 2.01 | 1.02 | 0.55 |
| 0.96 | 0.91 | 1.14 | 0.57 | 1.17 | 1.25 | 1.05 | 0.92 | 0.90 | 0.99 | 0.62 |
| 1.53 | 1.25 | 1.00 | 1.31 | 1.33 | 1.24 | 1.02 | 1.04 | 3.46 | 0.64 | 1.90 |
| 1.04 | 1.08 | 1.33 | 1.12 | 1.19 | 1.36 | 1.25 | 1.07 | 0.87 | 0.67 | 0.81 |
| 1.44 | 1.35 | 1.34 | 3.78 | 1.94 | 2.24 | 1.34 | 1.38 | 1.14 | 0.88 | 0.75 |

Supplementary figure 7.

Results of cytokine antibody array. The value in each box is calculated by the blot density readings of RAB31 CAFs CM/EGFP CAFs CM. Gray boxes are insignificant blots, which lacks credibility. Purple boxes are significantly down regulated cytokines; Orange boxes are significantly upregulated cytokines.


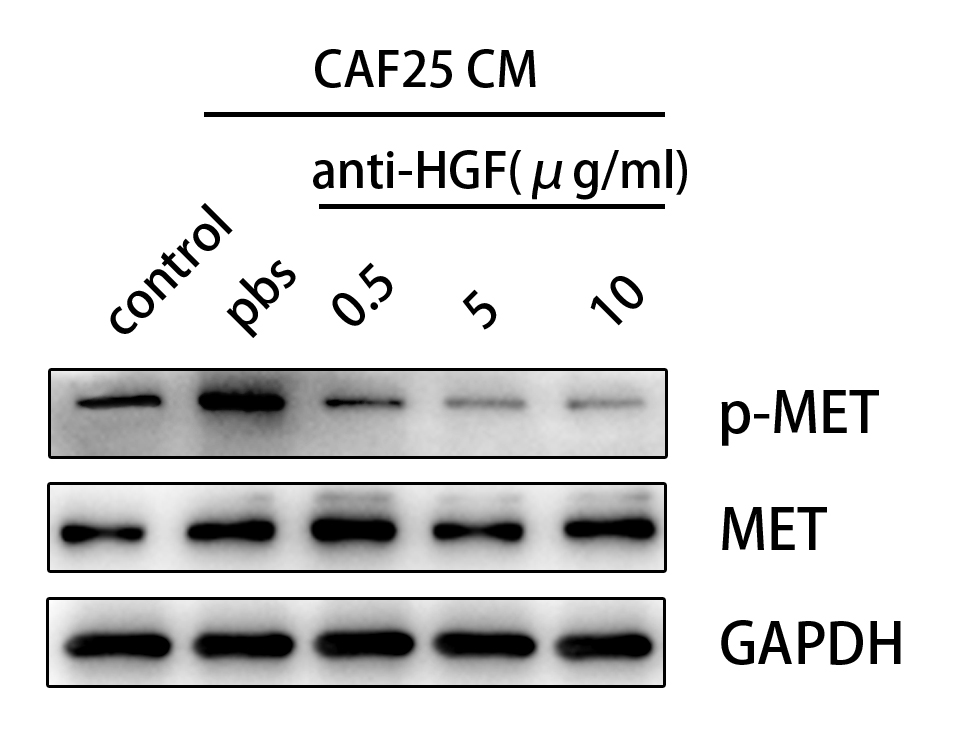


Supplementary figure 8.

Neutralization of HGF from CAF25 CM. WB show that a concentration of 5 μg/ml anti-HGF

polyclonal antibody reached maximum effect of inhibiting MET phosphorylation in RKO. Control

refers to RKO in culture medium at 2% FBS.

|  | **Primer sequence(5’-3’)** |
| --- | --- |
| **RAB31 overexpression** | **Forward** 5’- GCCTCGAGAATGATGGCGATACGGG - 3’ |
|  | **Reverse** 5’- GCGGATCCACAGCACCGGCGGCTGG- 3’ |
| **RAB31 Knockdown** | **Forward Oligo** 5’- CAGGTATGCACGCGTGAATTCGGAGTGCGACCTC  TCAGATATTACTCGAGTAATATCTGAGAGGTCGCACTCCTTTTTTGAATTCTCGACCTCGAGACAA-3’ |
|  | **Reverse Oligo** 5’-TTGTCTCGAGGTCGAGAATTCAAAAAAGGAGTGC  GACCTCTCAGATATTACTCGAGTAATATCTGAGAGGTCGCACTCCGAATTCACGCGTGCATACCTG-3’ |
| **Met Knockdown** | - **Forward Oligo** 5’-CAGGTATGCACGCGTGAATTCGCTGTGAGAATATAC - ACTTACCTCGAGGTAAGTGTATATTCTCACAGCTTTTTTGAATTCTCGACCTCGAGACAA-3’ |
|  | - **Reverse Oligo** 5’-TGTCTCGAGGTCGAGAATTCAAAAAAGCTGTGAG - AATATACACTTACCTCGAGTAAGTGTATATTCTCACAGCGAATTCACGCGTGCATACCTG-3’ |

**Table S1. Primers and Oligos used for Plasmid construction**

**Table S2. Primers for QPCR**

| **Genes** | **Primer sequence(5’-3’)** |
| --- | --- |
| **IL6** | Forward: 5’-ACTCACCTCTTCAGAACGAATTG-3’  Reversed: 5’-CCATCTTTGGAAGGTTCAGGTTG-3’ |
| **CXCL8** | Forward: 5’-TTTTGCCAAGGAGTGCTAAAGA-3’  Reversed: 5’-AACCCTCTGCACCCAGTTTTC-3’ |
| **HGF** | Forward: 5’-GCTATCGGGGTAAAGACCTACA-3’  Reversed: 5’-CGTAGCGTACCTCTGGATTGC-3’ |
| **IGF-1** | Forward: 5’-GCTCTTCAGTTCGTGTGTGGA-3’  Reversed: 5’-GCCTCCTTAGATCACAGCTCC-3’ |
| **CTGF** | Forward: 5’-CAGCATGGACGTTCGTCTG-3’  Reversed: 5’-AACCACGGTTTGGTCCTTGG-3’ |
| **RAB31** | Forward: 5’-GGGGTTGGGAAATCAAGCATC-3’  Reversed: 5’-GCCAATGAATGAAACCGTTCCT-3’ |
| **GAPDH** | Forward: 5’-AGAAGGCTGGGGCTCATTTG-3’  Reversed: 5’-AGGGGCCATCCACAGTCTTC-3’ |
